# Toxicological profile of the aqueous-fermented extract of *Musa paradisiaca *in rats

**Published:** 2018

**Authors:** Eziuche Amadike Ugbogu, Victor Chibueze Ude, Iheanyichukwu Elekwa, Uche Okuu Arunsi, Chikezie Uche-Ikonne, Chinedu Nwakanma

**Affiliations:** 1 *Department of Biochemistry Abia State University, PMB 2000, Uturu, Abia State, Nigeria*; 2 *Department of Medical Biochemistry, College of Medicine Enugu State University of Science and Technology, PMB 01660, Enugu-Nigeria*; 3 *Federal Medical Centre Umuahia, Abia State, Nigeria *

**Keywords:** Musa Paradisiaca, acute toxicity, Sub-acute toxicity, Haematology, Lipid profile, Histopathology

## Abstract

**Objective:**

This study was conducted to assess the toxicity profile of the aqueous-fermented extract of *Musa paradisiaca *in rats.

**Materials and Methods:**

In acute toxicity test, the rats of different groups were orally administered with a single dose of 500, 1000, 2000 and 5000 mg/kg of fermented extract of *M. paradisiaca*. The rats were monitored for behavioral changes, toxicity signs and mortality. In sub-acute test, the rats were orally administered with fermented *M. paradisiaca* extract (200, 400 and 800 mg/kg/day) for 14 days. Haematological and serum biochemical parameters were evaluated and histopathological studies of the liver and kidney were done. The study was performed from June to July 2017.

**Results:**

Concerning the acute toxicity, no toxicity signs or death were recorded and an LD_50 _value of >5 g/kg for fermented extract of *M*.* paradisiaca* was observed*.* Regarding the sub-acute toxicity, ingestion of the fermented extract of *M. paradisiaca* caused no significant effects (p<0.05) in terms of relative organ weight, body weight percentage, haemoglobin, red blood cells count, electrolytes levels, lymphocytes count, basophils count, and aspartate aminotransferase (AST) and alkaline phosphatase (ALP) levels. However, significant differences (p<0.05) were observed in white blood cells, eosinophils, platelets, neutrophils and monocytes counts, and urea, creatinine, alanine aminotransferase (ALT) and high-density lipoprotein (HDL) levels. The histological assessments of the liver and kidney showed normal results.

**Conclusion:**

The findings of this study has suggested that daily administration of fermented extract of *M. paradisiaca* at doses up to 800 mg/kg for 14 days, is not toxic and may be considered safe for therapeutic uses.

## Introduction

*Musa paradisiaca *(Linn.)* (Musaceae) *is a herbaceous plant which is commonly known as “plantain”. It is indigenous to South Asia and India and is presently grown in almost all tropical and subtropical regions of Africa including Nigeria. This plant grows up to 9 m in length and produces greenish or greenish-yellow seedless oblong fruits of up to 7 cm long in bunch form depending on the variety and the nutrient present in the soil (Dutta et al., 1993; Imam and Akter, 2011 ▶; Yakubu et al., 201 ▶). The proximate analysis of *M. paradisiaca *revealed the presence of protein, dietary fibre, carbohydrates, lipids, minerals such as potassium (K), magnesium (Mg), phosphorus (P), calcium (Ca), sodium (Na), zinc (Zn) and iron (Fe), and water-soluble vitamins such as thiamine, riboflavin, niacin, ascorbic acid and folic acid  (Ketiku 1973 ▶; Coulibaly et al., 2007 ▶; Eleazu et al., 2011 ▶; Ibukun et al., 2012 ▶; Adeolu and Enesi, 2013 ▶; Danlami et al., 2015 ▶; Kiin-Kabari and Giami, 2015 ▶; Annor et al., 2016 ▶).  The bioactive metabolites present in the fruits of plantain include, flavonoids, tannins, alkaloids, glycosides, phytates, oxalates, amino acids, steroids, benzoic acid derivatives, ascorbic acid, and vitamin A  (Dutta et al., 1983 ▶; Eleazu et al., 2011 ▶; Adeolu and Enesi, 2013 ▶; Rao et al., 2014 ▶; Danlami et al., 2015 ▶,) . 

Traditional medicine (TM) is comprised of explicable and inexplicable knowledge, acquired skills, and sometimes beliefs from different cultures of the world which are used in the treatment of diseases and maintenance of good health. TM is also designated as complementary and alternative medicine (CAM) and/or non-conventional medicine in developed countries (Zhang, 2000 ▶). In Africa, more than 80 % of the population use TM while in developed countries such as Canada, up to 70% of the population use CAM for their healthcare needs (World Health Organization, 2002 ▶) because of its accessibility, affordability, efficacy, bioavailability and less side effects (Elujoba et al., 2005 ▶; Ezekwesili et al., 2014 ▶; Elujoba et al., 2005 ▶; Ugbogu et al., 2016 ▶). 

Due to the presence of the several nutrients including minerals and bioactive metabolites in *M. paradisiaca*, virtually all parts of the plant have been used for management of various ailments. For example, aqueous extract of fermented unripe *M. paradisiaca* fruits and unripe *M. paradisiaca* peels possess anti-ulcerogenic (Ezekwesili et al., 2014 ▶; Ikpeazu et al., 2017 ▶) and anti-microbial (Kapadia et al., 2015 ▶) activities. Studies have also shown that aqueous extract of *M. paradisiaca *fruit pulp possesses antioxidant (Shodehinde and Oboh, 2013 ▶) and wound healing properties (Agarwal et al., 2009 ▶) and could be used as a remedy against diabetes and hepatic dysfunction (Ojewole and Adewunmi, 2003 ▶; Eleazu and Okafor, 2015 ▶). Extracts of *M. paradisiaca* stem are used for wound healing and have been shown to have antimicrobial activity (Amutha and Selvakumari, 2016 ▶) and hepato-protective property (Nirmala et al., 2012 ▶). *M. paradisiaca* flower possesses antimicrobial and anti-hyperglycemic (Jawla et al., 2012 ▶) as well as anti-diarrhoea effects (Yakubu et al., 2015 ▶). Amongst all these potential uses of *M. paradisiaca* for management and treatment of diseases, there is little or no published data on comprehensive assessment of the toxicity of aqueous extract of fermented *M. paradisiaca* which is currently used in treating ulcers. A previous investigation (Ikpeazu et al., 2017 ▶) revealed that the effectiveness of fermented products over the non-fermented counterparts is due to the synergistic role of phytoconstituents and microbiomes. Probiotics, as live microorganisms, have been observed to confer health benefits such as enhancement of mineral metabolism, reduction of LDL-C level, stimulation of vitamin B-complex biosynthesis, modulation of the immune system, anti-hypertensive and antiulcerogenic effects (Musa et al., 2009 ▶; Liong et al., 2009 ▶; Haukioja, 2010 ▶). It is against this backdrop that the current study was designed to assess the toxicity profile of aqueous-fermented extract of *M. paradisiaca *concerning body weight, relative organ weight, and histopathological indices as well as haematological, hepatocellular, and lipid profiles in rats.

## Materials and Methods

**Sample collection and identification**s

Fresh unripe plantain (*M. paradisiaca*) was purchased from Eke Okigwe Market, Okigwe Local Government Area in Imo State. It was authenticated by a Botanist as *Musa paradisiaca*; a sample was kept at the herbarium of University of Nigeria Nsukka with voucher number UNH No.: 812. 

**Sample preparation**

The rotten fruits were removed, and the rest were washed with clean water. *M. paradisiaca* were peeled, washed and cut into approximately 4-mm long pieces. With the aid of G & G® Electronic scale, 200 g of the sliced *M. paradisiaca* were weighed in a beaker and 300 ml of distilled water was added. The beaker containing the plantain and water was covered and allowed to stand at room temperature (25^o^C), overnight (i.e. from 6 pm to 8 am). After 15 h of fermentation, the extract was filtered using cheesecloth and the filtrate was used immediately.

**Animal handling**

A total of 74 healthy rats (150-200g) comprising 50 males and 24 female rats were purchased from University of Nigeria Nsukka and transported to Biochemistry Department, Abia State University Uturu, Nigeria. The rats were allowed to acclimatize for two weeks in a well-ventilated house with clean cages under normal environmental conditions of temperature (25-28^o^C) and humidity (35-60 %) with 12 h/12 h light/dark cycles, before the start of the experiment. The rats were fed with standard commercial food and they had free access to water. Strict adherence to ethical principles (Neuwinger 2000 ▶), of the World Health Organization of good laboratory practices and United States guidelines for animal experiments (Care, Animal, and Use Committee,1998 ▶, CNRC, 2010 ▶ was maintained in this study. Experimental procedures and animal handling were approved by the Abia State University Research Ethical Clearance Committee -ABSU/REC/BMR/015.

**Acute toxicity test (median lethal dose (LD **_50_ **)**

A total of 50 rats were divided into five experimental groups of ten (10) rats and each group consisted of five (5) female and five (5) male rats. The rats were fasted overnight and a single dose of 500, 1000, 2000 or 5000 mg/kg aqueous-fermented extract of *M. paradisiaca* fruit pulp was orally (i.e. gavage) administered to groups B, C, D and E, respectively while group A received 0.25 ml of distilled water and served as the control. The rats were monitored for behavioral changes, toxicity signs and mortality for 24 h and thereafter for 14 days (OECD, 2001) guideline 423 with little modifications. 

**Sub-acute toxicity study**

Twenty-four (24) male rats were allocated into four (4) experimental groups each consisted of six (6) rats. Aqueous-fermented extract of *M. paradisiaca *fruit pulp (200, 400, or 800 mg/kg) or 0.25 ml of distilled water for the control group, were administered once daily for 14 consecutive days (OECD, 1995 ▶) guideline 407.

**Organs and blood sample collection**

After 14-days administration of aqueous-fermented extract of *M. paradisiaca *fruit pulp the rats were anesthetized and sacrificed immediately on the 15^th^ day of the experiment*.* Blood samples were collected via cardiac puncture. The samples for biochemical tests were dispensed into heparinized containers while the samples for haematological analysis were collected into ethylenediaminetetraacetic acid (EDTA)-containers. The heart, kidney, liver, lungs and spleen were carefully removed by dissection and their weights were determined.

**Haematological studies**

The haematological studies were performed as described by    Bain et al. (2016)  ▶. The haematological parameters evaluated were haemoglobin (Hb) level, white blood cell (WBC) count, packed cell volume (PCV), red blood cell (RBC) count, platelet count, mean cell volume (MCV), mean corpuscular haemoglobin (MCH) and mean corpuscular haemoglobin concentration (MCHC). 

**Clinical chemistry studies**

Liver enzymes including alanine aminotransferase (ALT), aspartate aminotransferase (AST) and alkaline phosphatase (ALP); renal function parameters including bicarbonate (HCO_3_^-^), chloride (Cl^-^), creatinine, potassium (K^+^), sodium (Na^+^) and urea and lipid profile parameters including triglyceride, total cholesterol, high-density lipoprotein cholesterol (HDL-C), low-density lipoprotein cholesterol (LDL-C) and very low-density lipoprotein cholesterol (VLDL-C), were evaluated. All the above-mentioned parameters were evaluated using ready-to-use kits obtained from Randox Laboratory Ltd. Co. Antrim, United Kingdom based on the manufacturer’s instructions.

**Histological studies**

The liver and kidney were fixed in 10 % formalin after the rats were sacrificed, thereafter, samples were cleaved, processed and embedded in paraffin wax. Then, the tissues were sectioned into 5-m thickness specimen, stained with haematoxylin and eosin and evaluated under an optical microscope by an experienced pathologist as described by Fisher et al.         (2002)  ▶. 

**Statistical analysis**

The results of this study were presented as mean ± standard deviation of three replicates using excel package. The experimental data were tested for homogeneity of variance and then subjected to one-way analysis of variance (ANOVA) and the difference between the samples mean were tested by Tukey *post-hoc* test using R-statistics software version 3.03. A p≤0.05 was considered statistically significant. 

## Results

**Figure 1:**
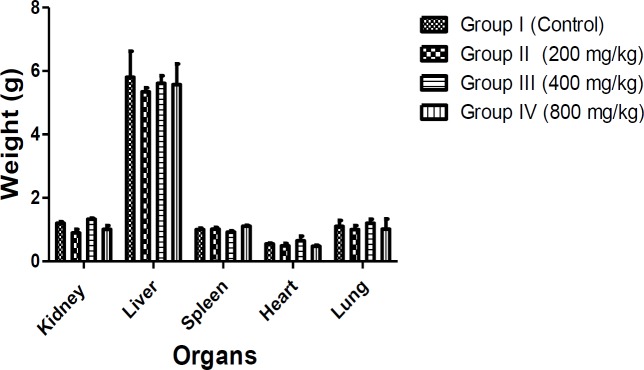
Effects of 14-days administration of aqueous-fermented *Musa paradisiaca* (200, 400 and 800 mg/kg body weight) on the relative organ weight of Wistar rats. Values represent mean ± SD of n=6 rats in each group. Significant differences compared to control at p<0.05 are indicated with *

In acute toxicity study, any signs of abnormalities before and after treatment with aqueous-fermented extract of *M. paradisiaca* fruit pulp (500-5000 mg/kg) were recorded. No death or signs of toxicity were observed in all the groups. However, rats fed with 2000 mg/kg extracts were calm within 2 h of administration while rats treated with 5000 mg/kg could not eat enough food (Table 1). In sub-acute toxicity test, no significant differences (p<0.05) were observed in percentage of weight gain and relative organ weight of all the groups (Table 2 and Figure 1). No significant differences (p<0.05) were observed in the blood profile in terms of Hb, PCV, MCH and MCV while significant differences (p<0.05) were observed in WBC and platelet counts of rats fed with 200, 400, 800 mg/kg of aqueous-fermented extract of *M. paradisiaca* compared to the control group (Table 3).

**Table 1:** Determination of acute toxicity (LD_50_) value of aqueous-fermented extract of *M. paradisiaca*

**Group**	**Dose (mg/kg)**	**D/T**	**Sign of toxicity/Behavioral changes**
**A**	0.25 ml (H_2_0)	0/12	No toxic effects
**I**	500	0/12	No toxic effects
**II**	1000	0/12	No toxic effects
**III**	2000	0/12	Calm, but agile after 2 h
**IV**	5000	0/12	Calm, agile after 2 h but could not eat enough food.

D/T = Number of rat deaths/Total number of rats used.

**Table 2:** The effects of aqueous-fermented* M. paradisiaca* on the body weight of Wistar rats post 14 days administration

**Parameter**	**Control**	**Group II (200 mg/kg)**	**Group III (400 mg/kg)**	**Group IV (800 mg/kg)**
**Weight on day 1**	116.00±9.54	107.67±16.44	159.67±3.79	108.67±7.51
**Weight on day 14**	150.60±13.58	141.00±47.13	182.67±6.66	130.33±10.79
**Weight gain (g)**	34.60	33.33	23.00	21.66

Values represent mean ± SD of n=6 rats in each group.

**Table 3:** Effects of aqueous-fermented extract of *M. paradisiaca* on haematological parameters of Wistar rats

**Parameter**	**Group I (control)**	**Group II (200 mg/kg)**	**Group III (400mg/kg)**	**Group IV (800 mg/kg)**
**PCV (%)**	49.40±1.70	46.90±0.95	45.17±0.51	44.80±2.67
**Hb (g/dl)**	13.90±1.10	12.70±1.18	13.70±0.30	14.53±0.38
**RBC (×10** ^12^ **/L)**	8.90±0.30	7.00±0.11	7.25±0.07	7.52±0.04
**MCV (fl)**	60.40±0.20	67.53±0.34	62.63±0.42	63.63±3.99
**MCH (pg)**	16.50±0.10	17.11±0.79	18.60±0.19	19.09±0.32
**MCHC (g/L)**	278.00±1.00	256.33±10.97	266.33±26.08	304.00±23.26
**WBC (x10** ^9^ **/L)**	17.00±1.30	10.90±0.75*	14.70±1.15	12.53±1.01*
**Lymphocyte (%)**	43.00±1.00	44.00±1.00	44.00±2.00	46.33±1.53
**Neutrophil (%)**	50.00±1.00	49.00±1.00	46.33±2.08*	42.33±2.52**
**Monocyte (%)**	4.80±1.00	4.67±1.15	7.33±3.06*	7.67±0.58*
**Eosinophil (%)**	1.67±0.58	1.67±0.58	3.00±2.00*	3.00±1.00*
**Basophil (%)**	0.00±0.00	0.00±0.00	0.00±0.00	0.00±0.00
**Platelet (×10** ^9^ **/L)**	555.00±50.23	581.67±54.85*	634.33±37.61**	722.00±41.62***

Values represent mean ± SD of n=6 rats in each group. Significant differences compared to control at p<0.05 are indicated with

*, **and *** showing the level of significance.

**Figure 2:**
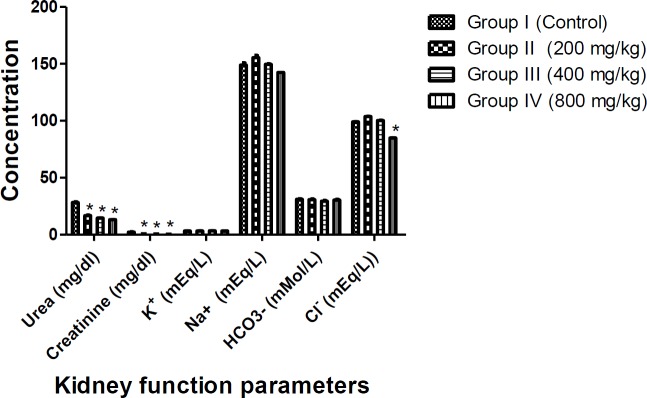
Effects of aqueous-fermented extract of *M. paradisiaca* on the kidney function parameters of Wistar rats. Rats were administered with aqueous-fermented extract of *M. paradisiaca* (200, 400 and 800 mg/kg body weight) for 14 days; then, rats were sacrificed and the kidney function parameters were assessed. Values represent mean ± SD of n=6 rats in each group. Significant differences compared to control at p<0.05 are indicated with *.

Significant decreases (p<0.05) in urea and creatinine levels were observed in the rats fed with aqueous-fermented extract of *M. paradisiaca* at all concentrations while Cl^- ^significantly reduced only at the dose of 800 mg/kg. No significant changes were observed in the serum levels of K^+^, Na^+^ and HCO_3_^-^ compared to the control group (Figure 2). 

**Figure 3:**
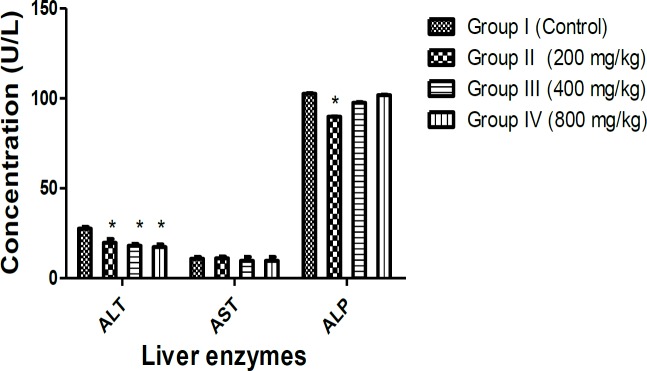
Effects of aqueous-fermented extract of *M. paradisiaca* on the hepatic enzymes of Wistar rats. Rats were administered with aqueous-fermented extract of *M. paradisiaca* (200, 400 and 800 mg/kg body weight) for 14 days; then, rats were sacrificed and the liver enzymes were assessed in serum. Values represent mean ± SD of n=6 rats in each group. Significant differences compared to control at p<0.05 are indicated with asterisk *.

ALT showed a significant decrease in rats treated with aqueous-fermented extract of *M. paradisiaca* at all concentrations while ALP value ranged from 89.80±0.17 to 102.50±0.40 U/L and AST ranged from 9.67±2.08 to11.00±1.00 U/L (Figure 3).

**Figure 4:**
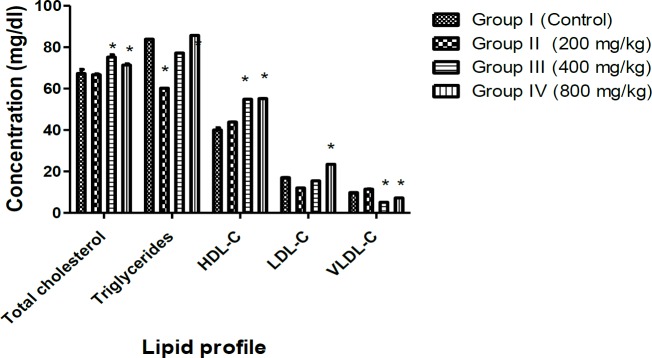
Effects of aqueous-fermented extract of *M. paradisiaca* on lipid profile parameters of Wistar rats. Rats were administered with aqueous-fermented extract of *M. paradisiaca* (200, 400 and 800 mg/kg body weight) for 14 days; then, animals were sacrificed and the lipid profile was determined in serum. Values represent the mean ± SD of n=6 rats in each group. Significant differences compared to control at p<0.05 are indicated with *.

**Figure 5:**
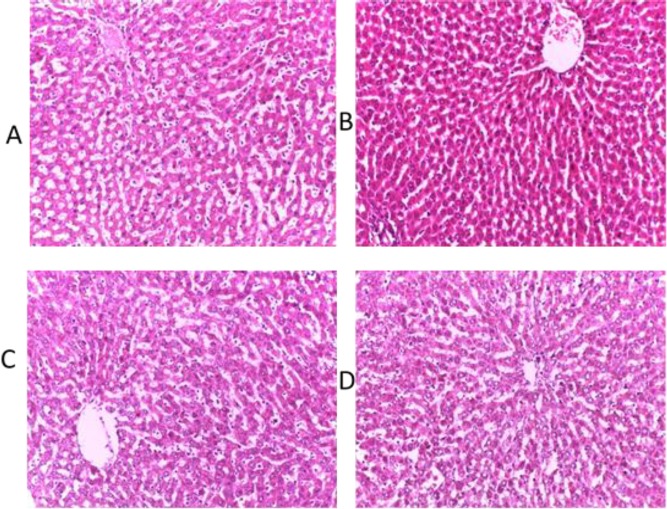
Micrographs of the liver sections obtained from untreated (control) rats and rats treated with various doses of aqueous-fermented extract of *M. paradisiaca* fruit pulp. Haematoxylin and eosin staining (H&E), magnification (40X). (A) control; (B) Wistar rats treated with 200 mg/kg aqueous-fermented extract of *M. paradisiaca*; (C) Wistar rats treated with 400 mg/kg aqueous-fermented extract of *M. paradisiaca*; and (D) Wistar rats treated with 800 mg/kg aqueous-fermented extract of *M. paradisiaca*

**Figure 6:**
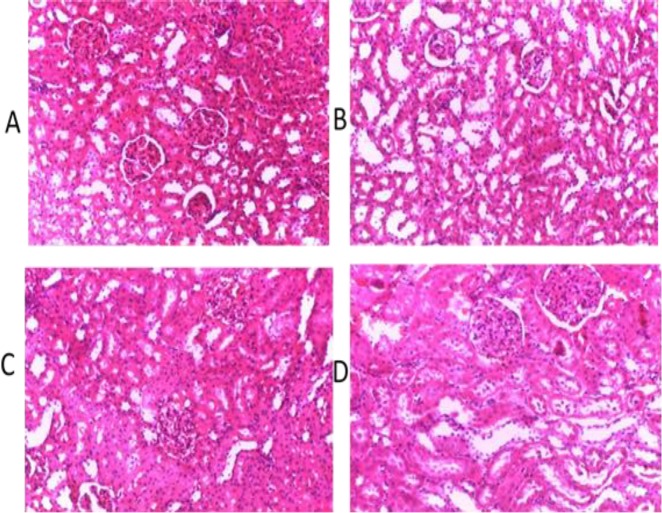
Micrographs of the kidney sections obtained from untreated (control) rats and rats treated with various doses of aqueous-fermented extract of *M. paradisiaca*. Haematoxylin and eosin staining (H&E), magnification (40X). (A) control; (B) Wistar rats treated with 200 mg/kg aqueous-fermented extract of *M. paradisiaca*; (C) Wistar rats treated with 400 mg/kg aqueous-fermented extract of *M. paradisiaca* and (D) Wistar rats treated with 800 mg/kg aqueous-fermented extract of *M. paradisiaca*

Lipid profile investigation showed that cholesterol, LDL-C and HDL-C increased significantly (p<0.05) in groups treated with 400 and 800 mg/kg extract of *M. paradisiaca* compared to the control group (Figure 4). There were no observable changes in histology of the rats’ liver and kidney (Figures 5 and 6).

## Discussion

In folk medicine, various parts of *M. paradisiaca* are used in the management or treatment of many ailments such as diabetes, diarrhea, burns, hypertension, marasmus, bites, hemorrhage and ulcers.    Ikpeazu et al. (2017)  ▶ established that aqueous-fermented extract of *M. paradisiaca* has an effective anti-ulcer potential. However, regardless of various medicinal uses of *M. paradisiaca*, there is little or no comprehensive report on the toxicity profile of aqueous-fermented extract of *M. paradisiaca *fruit pulp. Aqueous-fermented extract of *M. paradisiaca *fruit pulp is used in Southeastern parts of Nigeria to treat ulcers. However, administration of this extract over a long period of time may be detrimental in the absence of proper dose monitoring or without considering its toxic effects. This study therefore investigated acute and sub-acute toxicity profile of aqueous-fermented extract of *M. paradisiaca* in rats. 

Toxicity studies of substances involve acute, sub-acute, chronic, or sub-chronic toxicity (Balogun and Tom Ashafa, 2016 ▶; Kong et al., 2016b ▶) . In acute toxicity studies, oral administration of fermented *M. paradisiaca* 500 and 5000 mg/kg to rats did not cause mortality nor toxicity signs. However, behavioral changes were observed at the doses of 2000 mg/kg (rats were calm within 2 h) and 5000 mg/kg (rats lost appetite for food). This is an indication that the LD_50_ of *M. paradisiaca *extract is well above 5000 mg/kg and an LD_50_ of >5000 mg/kg has been reported to be safe and can be considered non-toxic (OECD, 2001 ▶). Determination of organ weight in *in vivo* toxicity studies is essential for assessment of animals’ sensitivity to toxicity, physiologic perturbations, induction of enzymes, and acute organ damage (Michael et al., 2007 ▶) . In sub-acute toxicity studies, non-significant differences (p<0.05) in weight gain (Table 2) and relative organ weight (Figure 1) were observed in all groups demonstrating normal weight increase in all groups.

Blood is regarded as an essential biological sample for assessment of physiological, nutritional and pathological status of vertebrates. Investigation of blood parameters are also useful for determination of the impact of potentially toxic substances on blood parameters (Han et al., 2010 ▶) . Significant increases (p<0.05) which were within the internationally accepted range of the proportions of neutrophils, eosinophil and monocytes and non-significant changes in lymphocytes and basophils counts suggest that this extract did not cause any damage to the tissues nor the immune system. Significant increases in platelet counts observed in all extract-treated groups compared to control group, are possibly because of elevated secretion and production of thrombopoietin, a hormone responsible for the synthesis of platelets (Kaushansky, 1995 ▶) . Hepatic and renal functions impairment caused by toxicants could be revealed by investigation of blood and biochemical parameters as liver and kidney are pivotal for survival (Olorunnisola et al., 2012 ▶) . ALP, AST and ALT are essential biomarkers of cellular integrity and function of the liver and heart, which are often released into the blood from damaged liver (Chavda et al., 2010 ▶) . Cellular damage, tissue necrosis and cardiovascular diseases lead to elevation of serum concentrations of ALT and AST (Ioannou et al., 2006 ▶; Adeyemi et al., 2015 ▶) . In this study, serum levels of ALT significantly reduced (p<0.05) while AST slightly decreased, suggesting that aqueous-fermented extract of *M. paradisiaca* may possess some hepato-protective properties. 

Increases or decreases in serum electrolytes level may be caused by a hypo- or hyper-functioning organ or tissue. Kidney functions are commonly investigated by assessing the level of sodium, potassium, and chlorides in blood serum (Balogun and Tom Ashafa, 2016 ▶) . The serum concentration of urea and creatinine decreased significantly (p<0.05) while HCO^-^_3_, K^+^ and Na^+ ^levels were not significantly different from those of the control group (p<0.05), indicating that *M. paradisiaca* has no adverse effect on the kidney. Although, significant increases (p<0.05) were observed in total cholesterol, HDL-C and LDL-C levels, the observed increases in values were within the normal range. The histological assessment of the liver and kidney showed normal architecture of the organs without any detrimental pathological changes confirming non-toxic nature of *M. paradisiaca* at tested doses (Figures 5 and 6).

In conclusion, this study revealed that the LD_50 _of aqueous-fermented extract of *M. paradisiaca *is above 5g/kg. The study also established that the oral administration of aqueous-fermented extract of *M. paradisiaca* up to the dose of 800 mg/kg for 14 days, is not toxic and therefore may be considered safe for therapeutic uses. This study also revealed that aqueous-fermented extract of *M. paradisiaca *decreased liver biomarker enzymes (e.g. ALT, AST and ALP) when compared to their respective controls, suggesting that aqueous-fermented extract of *M. paradisiaca *could have hepato-protective effects. 
